# Investigation into the role of phenolic compounds in the protection of citrus against *Phyllosticta citricarpa*

**DOI:** 10.1007/s11306-024-02095-1

**Published:** 2024-02-26

**Authors:** Melida Mabogoane, Wilma Augustyn, Vuyelwa J Tembu, Thierry Regnier, Wilma du Plooy

**Affiliations:** 1 Department of Chemistry, Tshwane University of Technology, Private Bag X680, Pretoria, 0001 South Africa; 2 Department of Biotechnology and Food Technology, Tshwane University of Technology, Private Bag X680, Pretoria, 0001 South Africa; 3 Citrus Research International, P.O. Box 28, Nelspruit, 1200 South Africa

**Keywords:** Citrus black spot, *Phyllosticta citricarpa*, Biomarkers, Tolerance, Phenolic compounds

## Abstract

**Introduction:**

The use of chemical fungicides to combat disease has made a substantial contribution to food quality and security. Nonetheless, their applications have been limited due to environmental and health concerns, unaffordability, and the fact that pathogens have acquired resistance to some of these fungicides. Alternative eco-friendly and safe control methods should be explored. The current study investigated the influence of citrus rind phenolic compounds against *Phyllosticta citricarpa* infection by metabolic profiling of two citrus cultivars with varying degrees of susceptibility to infection.

**Methods:**

Chromatographic data obtained by Ultra Performance Liquid Chromatography-Mass Spectrometry (UPLC) was subjected to multivariate data analysis to identify biomarkers associated with the tolerant cultivar. The identified biomarkers were tested in vitro against *P. citricarpa.*

**Results:**

Seville oranges, a tolerant cultivar, displayed higher levels of phenolic content and lower total sugar content, that are both associated with lower susceptibility to citrus black spot infection. The generated Principal Component Analysis (PCA) and Orthogonal Projection to Latent Structures-Discriminant Analysis (OPLS-DA) models gave an overview of the data set and identified components that may be responsible for the differences in susceptibility between the two cultivars. Candidate biomarkers associated with tolerance were identified as naringin, neoeriocitrin, bruteiridin, melitidin, and lucenin-2.

**Conclusion:**

Naringin, a major candidate biomarker was able to inhibit the growth of the pathogen at 10 000 ppm.

## Introduction

Citrus fruit is a member of the Rutaceae family, consisting of 140 genera and 1600 species (Chavan et al., 2018). The fruit is an important agricultural crop due to its high annual consumption and trade (Chen et al., 2020: Ho and Kuo, 2014). Citrus fruit is widely cultivated in over 140 countries throughout the world’s tropical and subtropical regions (Ladaniya, 2008). Citrus fruit is mainly produced in the United States of America (USA), Brazil, China, Argentina, Australia, Cuba, India, Israel, Italy, Japan, Mexico, Morocco, South Africa (SA), and Spain (Gill, 1997). In SA, citrus is cultivated in several regions: Limpopo, Mpumalanga, Kwa-Zulu Natal, the Eastern Cape, Western Cape, and the Northern Cape. Globally, SA is ranked the 10th largest producer of fresh citrus fruit and the second largest exporter of fresh citrus fruit in the world, with close to 1.7 million tons exported (Zhong and Nicolosi, 2020).

The South African citrus industry produces more than two million tons of fresh citrus a year, of which 65% is traded in foreign markets, 29% is supplied to the domestic market, and the outstanding quantities are sold to processing industries. The USA, Asia, the Middle East, Russia, and the European Union are the most important export markets for South African citrus fruit (Dlikilili and van Rooyen, 2018). Citrus exporters have taken advantage of favourable market conditions, and the demand for vitamin C-rich fruit has increased, particularly during the current Covid-19 pandemic. In 2021, 247 282 citrus pallets were shipped to the European continent from South Africa, compared to 171 704 and 209 291 during the same period in 2020 and 2018, respectively. Citrus cultivation is crucial to the South African economy, not only for its contribution to the Gross Domestic Product (GDP) but also because it employs many seasonal and permanent workers (Avhurengwi and Kena, 2017). However, the sector has been plagued by phytosanitary issues, particularly diseases, which have had a significant impact on citrus fruit production and trade over the years. The majority of commercial citrus cultivars are susceptible to the fungal disease known as Citrus Black Spot (CBS), which is caused by the ascomycete *Phyllosticta citricarpa* (Yonow et al., 2013). The infection results in cosmetic lesions affecting the local and international trade of citrus fruit. The management of CBS is currently reliant on the extensive use of chemical fungicides, which are toxic and expensive, and the pathogen has already developed resistance against some fungicides such as Benomyl. Moreover, concerns were raised about the dangers that chemical fungicide residues cause to human health, as well as environmental concerns about their disposal. Maintaining the different export markets requires compliance with specific standards set by each market (European Union, 1998 and, 2006) and South African citrus producers spend between almost R4 billion annually to comply with the phytosanitary regulations of the European Union (Esterhuizen & Caldwell, 2022).

Despite the citrus family’s well-researched pharmacological effects, there is limited data on the physiological and biological characteristics of citrus plants’ defense responses to environmental challenges and/or plant diseases such as CBS. Moreover, the relationship between secondary metabolites produced by citrus fruits and their role in CBS protection remains unclear. Citrus fruits were found to contain a large number of phenolic compounds, many of which have biological, antifungal, and antibacterial properties (Blount et al., 1992; Dai et al., 1996; Mierziak et al., 2014). These compounds are synthesized, in many cases, by induced systematic resistance in response to pathogen/disease exposure (Lattanzio et al., 2006). In this study, the phytochemistry of two citrus types with different susceptibilities to CBS was investigated. Seville oranges are tolerant and Eureka lemons are highly susceptible to CBS infection (Miles et al., 2019). Metabolic profiling revealed candidate biomarkers that play a role in protection and defence against pathogen infection and provide environmentally friendly alternative strategies for fruit quality control. Bioactive compounds with antifungal activity against *P. citricarpa* could lead to the development of environmentally friendly natural fungicides and assist with the cultivation of citrus fruit with high antifungal activities.

## Materials and methods

### Sampling of citrus varieties

The rinds of the following citrus varieties: Eureka lemons and Seville oranges were sampled from Mpumalanga in South Africa. The fruit was collected from fruit set until they were fully developed, at regular intervals over two full growing seasons. The citrus cultivars used in this study were selected based on the difference in their degree of susceptibility to citrus black spot. The rinds of the citrus cultivars were stored at – 80 °C before freeze-drying and extraction.

### Standards, solvents and chemicals

The solvents, acids, and chemicals used were AR grade and supplied by SMM Instruments (South Africa), except for acetonitrile used in HPLC separations, which was HPLC grade and obtained from Labscan (South Africa). All phenolic standards were purchased from Sigma-Aldrich (South Africa) or Industrial Analytical (South Africa) in the purest forms available. Fungicides used were Demildex, MHTCO_3_ and cuprous oxide obtained from Delta Chemicals, (Meyerton, South Africa), Agri-Cure SP (Delmas, South Africa) and Nordox 86%, WG Avima, (Kenmore, South Africa), respectively.

### Microwave-assisted extraction of non-volatile secondary metabolites in citrus fruit rind

Microwave extraction of rind secondary metabolites was done according to the method reported by Augustyn et al. (2014).

### Determination of total sugar content

The total sugar content (TSC) in the peel extract was determined using the phenol-sulphuric acid method. Briefly, the extracted phenolic compounds (Sect. 3.3), were diluted 50 times using distilled water. A test tube was filled with 500 µL extract, 500 µL freshly prepared phenol (5%, V/V), and 2.50 mL of sulphuric acid (99.9%). The resulting solution was mixed and incubated at 30 °C for 30 min. An aliquot of the test tube solution was then transferred in triplicate into a 96-well microtitre plate. A Spectramax190 microplate reader (Molecular Devices, Sunnyvale, USA), was used to measure the absorbance of the samples at 520 nm. Glucose standard solutions ranging from 1 to 5 mg/mL were prepared, and a standard curve was constructed and used to determine the total sugar content of the extracts, expressed as mg glucose/g dried peel (DP).

### Determination of total phenolic content

The quantity of phenolic compounds present in the citrus peels was determined using the spectrophotometric Folin-Ciocalteau method, as described by Mcharek & Hanchi, (2017). The concentration of the phenolic compounds was calculated and expressed as gallic acid equivalents (GAE) per gram of dry peels using a calibration curve (standard gallic acid solutions ranging from 0.1 mg/mL to 0.5 mg/mL).

### Ultra-performance Liquid Chromatography – Mass Spectroscopy

A Waters Acquity Ultra Performance Liquid Chromatographic-Xevo G2 Q-TOF system (Waters, Milford, MA, USA), equipped with a binary solvent manager (BSM), an autosampler with a fixed loop configuration (FL) and photodiode array detector (PDA), was used to profile secondary metabolites from the peels of the two citrus cultivars under investigation.

MassLynx® 4.1 software (Waters, USA) was used to collect data from the PDA detector and the Q-TOF mass analyser. Separation was accomplished using an Acquity UHPLC BEH C18 column (150 mm x 2.1 mm i.d. and 1.7 μm particle size) controlled at 40 °C. The mobile phase was 0.1% formic acid in water (solvent A) and acetonitrile (solvent B) at a flow rate of 0.30 mL/min, and the gradient as follows: 95:5 (A: B) at time zero then changed to 65:35 (A: B) over 10 min and held at this ratio for 3 min before returning to 95:5 (A: B), and a post-run time of 1.5 min. The total run time was 15 min. The injection volume was 2.00 µL. Mass spectrometry was carried out in the negative ionization mode. Nitrogen gas was used as the desolvation gas at a flow rate of 500 L/Hr while maintaining a desolvation temperature of 300 °C. The source temperature was 100 °C. Capillary and cone voltages were held constant at 2500 and 40 V, respectively, and the data were collected over the range *m/z* 100 to 1200 and centroided during acquisition using independent reference lock-mass ions (Leucine encephalin, 554.2615 *m/z)* infused through the fluidics system of the mass spectrum. This was performed to ensure mass accuracy and reproducibility.

MarkerLynx® 4.1 (Waters Corporation, Milford, MA, USA) software was used to pre-process the UPLC-MS chromatographic data. Pre-processing included peak detection, baseline correction, noise removal, and spectral alignment, which involved aligning molecular fragments with corresponding retention times over the entire data set. The aligned data were then exported to an Excel worksheet, before being subjected to multivariate data analysis using SIMCA-P®+ 14. The quality control (QC) sample which was a mixture of all samples, was run in duplicate after every 25 extracts analysis. Overlapping display analysis of the total ion flow diagram of mass spectrometry of different QC samples can be used to determine the repeatability of metabolite extractions.

### Multivariate data analysis

Comprehensive analysis of the UPLC-QToF-MS data was performed using multivariate data analysis tools. Pareto (par) scaling was applied to the chromatographic data. Chemical variation between the citrus cultivars was investigated through supervised and unsupervised chemometric models such as principal component analysis (PCA) and orthogonal projections to latent structures-discriminant (OPLS-DA).

Principal component analysis was performed to investigate the patterns in the data matrix and identify similarities or differences in chemical constituents among the cultivars based on the chromatographic data. OPLS-DA was used to correlate the CBS-resistant activity of Seville oranges to their phenolic compound constituents. The OPLS-DA model was validated by obtaining the ROC (Receiver operating characteristic) plot, permutation plot and CV-ANOVA (Cross validated analysis of variance). Subsequently biomarkers responsible for the activity was determined by an OPLS-DA S- plot. The chromatographic data was grouped into two classes according to the susceptibility levels, with Class 1 (Seville extracts - tolerant cultivar), and Class 2 (Eureka lemon extracts - highly susceptible cultivar).

### Identification of biomarkers

The UPLC–QToF–MS/MS technique was employed to analyse samples that contained biomarkers outlined by the OPLS-DA loadings plot. The analysis was carried out using the method described in Sect. 3.6 at higher collision energy and with a focus on molecular ions/peaks of interest. The outlined candidate biomarkers were represented as deprotonated ions ([M-H]^−^) and compound identifications were subsequently performed by comparing the UV spectra and mass fragmentation pattern to those of compounds reported in the literature and online databases such as Metlin.

### In vitro evaluation of naringin and fungicides against *Phyllosticta citricarpa*

#### *Phyllosticta citricarpa* mycelia production

A pure culture of *Phyllosticta citricarpa* was provided by the Citrus Research Institute (Nelspruit, South Africa). The isolate was sub-cultured and maintained on Potato Dextrose Agar (PDA) (Oxoid, Johannesburg, South Africa) in a temperature-controlled environment at 27 ± 2 °C for seven days prior to the experiment. Carrot sucrose agar (CSA) media was prepared according to the method described by Peres et al. (2007), with minor modifications. Briefly, 10.00 g of carrot puree, 3.50 g of sucrose, and 1.00 g of bacteriological agar in 50.00 mL of distilled water were autoclaved for 15 min. The agar was then poured into a sterilized petri dish and allowed to set. A 5 mm diameter agar plug containing active mycelia was aseptically collected from the edge of each actively growing fungal colony and placed at the centre of CSA plates. The plates were sealed with parafilm and incubated at 27 °C ± 2 °C for three weeks.

#### In vitro antifungal properties of naringin and selected fungicides

This study was conducted on one candidate biomarker (naringin) identified in Seville oranges (the tolerant cultivar). A 20 000 mg/L stock solution of naringin was prepared and the in vitro exposure to 1 000, 3 000, 4 000, 5 000, 7 500 and 10 000 mg/L concentrations of naringin was used to evaluate the antifungal properties of the candidate biomarker. In addition, three fungicides, Demildex, MHTCO_3_ and cuprous oxide, were tested at 2000 mg/L, 10 000 mg/L and 18 000 mg/L respectively and served as positive controls while water was used as negative control. The CSA media and inoculation were performed as mentioned in Sect. 3.9.1. For each compound, 10 replicates of each concentration tested were prepared. After 14 days of incubation at 27 °C ± 2 °C, mycelial growth was measured (in mm) with a digital calliper (Absolute Digimatic-Mitutoyo Corp. Japan). Percentage inhibition of mycelial growth was determined as described by Plaza et al. (2004).

### Statistical analysis

All TSC, TPC, and LC-MS experiments were carried out in triplicate. The data were expressed as the mean ± SD of three independent experiments performed in triplicate. Microsoft Excel® 2013 software was used for all statistical analyses.

## Results and discussion

### Total sugar content

Most of the studies on sugar content performed during the fruit development of citrus cultivars indicated an increase in total sugar content (TSC) throughout the fruit developmental stages (Albertini et al., 2006). According to Liu et al. (2012), sugars increase with fruit maturation and contribute 30–50% of the peels’ dry weight. In all data gathered for this assay, the cultivars indicated a similar trend where the ripe/mature stage indicated higher sugar content than at the beginning of the fruit set (green/small), Table 1. Seville oranges and Eureka lemons displayed similar trends in TSC values for all developmental stages. The TSC value of Seville oranges at green and ripe stages was lower than that of the Eureka lemon values, but higher for the colourbreak stage. CBS infection takes place in very young fruit and the lower sugar levels observed for Seville oranges may play a role in the tolerance of this cultivar.

According to Katz et al. (2011), an increase in sugar content occurs during citrus fruit maturation when the organic acid and amino acid accumulation seen during the early stages of growth transition into sugar synthesis during the later stages of development.

### Total phenol content

The difference in total phenolic contents (TPC) between the peel extracts of the two cultivars is reported in Table 1.

**Table 1:** Total phenolic and total sugar content of the peel extracts of Seville oranges and Eureka lemons, at different fruit development stages

	TSC (mg/g dry peel)			TPC (mg GAE/g DP)		
	G	CB	R	G	CB	R
Seville	71.28 ± 1.8	98.9 ± 1.2	231.3 ± 0.8	189 ± 0.4	78.2 ± 1.8	98.3 ± 1.3
Lemons	115.4 ± 1.4	79.9 ± 0.4	249.2 ± 1.1	69.5 ± 0.9	41.1 ± 1.6	31.8 ± 1.2

Data represent the mean ± SD of three samples analysed separately. G = Green fruit stage, CB = Colourbreak stage, R = Ripe stage

The total phenolic content varied between the cultivars during fruit developmental stages, ranging from 189 to 31.8 mg GAE/g DP. Seville oranges produced the most soluble phenolics (189 mg GAE/g DP) at the beginning of the fruit set when citrus fruit are more susceptible to CBS infection. Eureka lemons produced less phenolics at this stage (69.5 mg GAE/g DP).

Phenols and their derivatives play an important role in plant pathogen defense (Al-Qassabi et al., 2018). In this study, the phenol content was higher in Seville oranges, the more tolerant cultivar. The cultivar most susceptible to CBS, Eureka lemons, displayed lower phenol values, Table 1. This may be correlated to the difference in susceptibility of these cultivars to the CBS pathogen. Interestingly, a higher phenol content of 75.30 mg/g was observed in peel extracts of CBS-infected Eureka lemons as compared to the other Eureka lemon extracts. According to Naczk and Shahidi (2006), plants synthesize secondary metabolites as they grow, as well as in response to pathogen infection, wounding, and exposure to UV radiation and extreme temperatures. These metabolic actions explain why CBS-positive rinds exhibited higher phenol content than uninfected peels. As reported by Ayaz et al. (2008), the decrease in phenolic compounds during citrus fruit development could be attributed to the increased activity of polyphenol oxidase due to a rise in fruit pH as well as the reduced activity of phenylalanine ammonia-lyase.

### Ultra-performance liquid chromatography

**Fig. 1:**
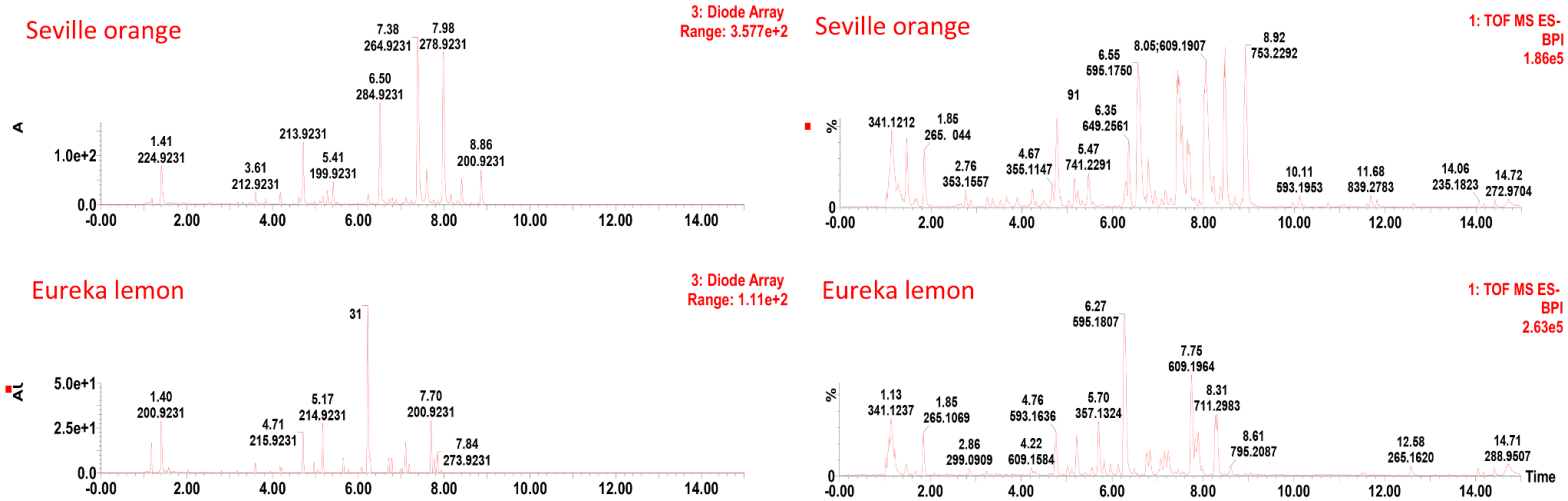
UPLC-UV (left) and UPLC-MS (right) chromatograms indicating the variation in phenolic constituents between rind extracts of Seville oranges and Eureka lemons, at mature/ripe stage of fruit development

UPLC-DAD-MS was employed to investigate specific metabolites responsible for the differences observed in the phenolic content indicated in Table 1. The data followed the same trend observed with TPC. More individual metabolites and higher levels were detected at the start of the fruit set (green stage), decreased at colourbreak, and increased slightly at a mature fruit stage. Detection by DAD and MS indicated that the two citrus cultivars contained different major compounds, Fig. 1. It should be noted that Seville oranges contained a high number of major compounds compared to that of the Eureka lemons, Fig. 1.

### Multivariate data analysis

Chemometric modeling of UPLS-QToF-MS data using multivariate data analysis was employed to assess and characterize phytochemical differences between the peel extracts of Seville oranges and Eureka lemons, using an untargeted metabolomics approach.

**Fig. 2:**
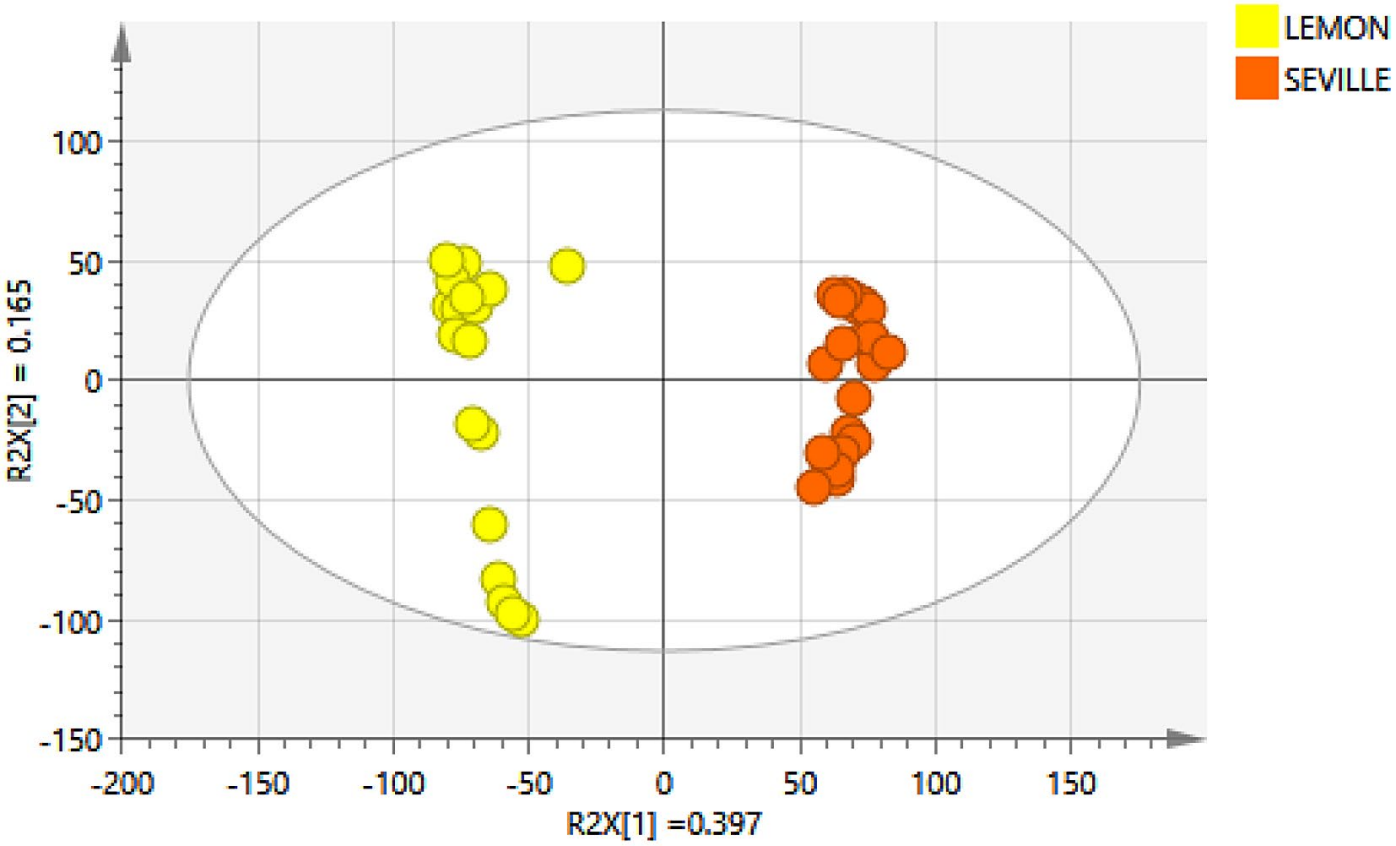
PCA scores plot in Pareto scale, clearly demonstrating separation between Eureka lemons (highly susceptible, in yellow) and Seville oranges (tolerant, in orange)

The principal component analysis (PCA) scores plot with good model parameters, R^2^X (cum) = 0.868 and Q^2^ (cum) = 0.672, displayed in Fig. 2, revealed a distinct separation between the two citrus cultivars. The two distinct clusters that can be seen in the PCA score plot indicated that the investigated metabolites had diverse chemical profiles. The model statistics revealed that 0.397 of the variation was modeled along PC1 and 0.165 was modeled along PC2, which contributed to the observed separation. According to the statistical data, these phenolic compounds are separated by significant chemical information. It can therefore be concluded that samples are strictly clustered according to cultivars, indicating differences in the chemistry of samples from different citrus species.

The variation in the metabolite profile that was observed with PCA plots led to further investigation of the two cultivars with varying susceptibilities to CBS. This allowed the identification of biomarkers responsible for the observed lower susceptibility of Seville oranges against the *Phyllosticta citricarpa* pathogen. The supervised orthogonal projections to latent structures discriminant analysis (OPLS-DA) was performed. This method can be used for the identification of significant variables responsible for the differentiation of these classes.

**Fig. 3:**
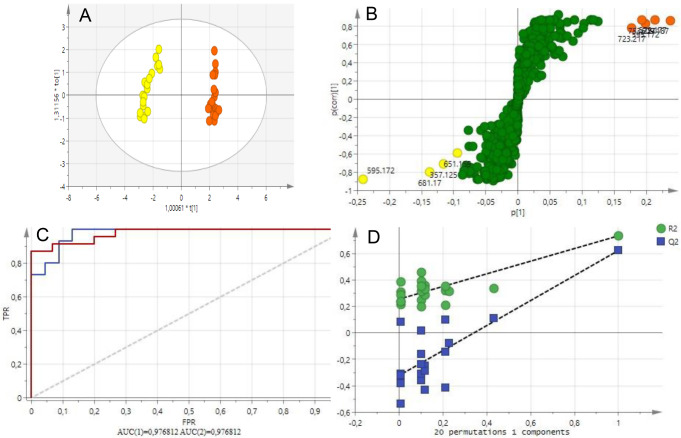
OPLS-DA plots. **A**: Scores plot indicating a clear separation between the peel extracts of Seville oranges and Eureka lemons, **B**: S-plot indicating the biomarkers with the highest contribution to the separation, marked with *m/z* values, **C**: ROC plot and D: Permutation plot

The obtained OPLS-DA scores plotted in Fig. 3A, similar to PCA analysis, displayed a good separation based on classes. The R^2^X of the OPLS-DA model indicated 0.550 of the variation in the dataset could be modeled by the selected components. The R^2^Y of the model was 0.734 indicating that the model was reliable. The Q^2^ was 0.623 indicating good predictability of the model.

The OPLS-DA S-plot, Fig. 3B, depicts the phytochemical relationships between the extracts, from Seville orange and Eureka lemon, and the compounds detected by UPLC X QToF-MS. Biomarkers can be identified through S-plots derived from OPLS-DA models. S-plots are used to provide a good overview of the data and model. The S-plot visualizes both the covariance and correlation between the metabolites and the modelled class designation (Wiklund et al., 2008). The p(corr) axis represents the reliability of each variable and has a value between + 1 and − 1. Variables at the extreme lower left and upper right quadrants have a low chance of inaccurate correlations, and therefore, were selected as putative markers. The predictability of this model was validated by the ROC plot, Fig. 3C. The ROC plot displays the true positive classification rate (TPR) plotted against the corresponding false positive classification rate (FPR). The area under the (ROC) curve (AUC) values (0.976812) indicate a reliable classification of biomarkers. The permutation curve also confirmed the validity of the OPLS-DA model, Fig. 3D. All Q2 values from the data set to the left are lower than the Q2 value on the actual data set to the right and the regression line has a negative value of intercept on the Y-axis. The model significance was confirmed by CV-ANOVA test where *P* < 0.05 (1.156 × 10^− 6^) indicates a reliable model.

This result indicated a difference in secondary metabolite composition between these cultivars, which may be connected to the difference in the degree of susceptibility of these citrus fruits to CBS infection. The S-plot of the OPLS-DA model indicated candidate biomarkers primarily responsible for the phytochemical difference observed among the two cultivars. These candidate biomarkers were marked in orange for Seville oranges (5 biomarkers), and yellow for Eureka lemons (4), Fig. 3B.

#### Identification of candidate biomarkers from Seville orange fruit

Structure elucidations were achieved using the following mass fragmentation guidelines: The loss of 162 Daltons indicated hexose (glucose or galactose, the most common sugars found in flavonoids), the loss of 146 Daltons indicated rhamnose, the loss of 133 Daltons indicated pentose (xylose or arabinose), and the loss of 308 Daltons indicated compounds with the disaccharide structure rutinose or neohesperidose linked through an *O*-glycosidic bond (Abu-Reidah et al., 2013). Moreover, the key fragmentations used in the MS identification of C-glycosides were [M-60]^−^, [M-90]^−^, [M-120]^−^, and [M-240]^−^ (Abad-García et al., 2012).

**Biomarker 1**: The compound with a UV wavelength of 273 nm, eluted at 7.4 min, with a [M-H]^−^ peak at 579.2 and the [2 M-H]^−^ precursor ion at 1159.4537 was tentatively identified as naringenin 7-*O*-neohesperidoside (naringin). The fragmentation ion, at m/z 459.1814 corresponds to the loss of a terminal C-glucosyl moiety [M-H-120]^−^, the fragmentation ion at m/z 271.1123 relate to the loss of a neohesperidose moiety [M-H-308], forming the unbound of naringenin ion, (Abu-Reidah et al., 2013; Brito et al., 2014; Guccione et al., 2016; Sánchez-Rabaneda et al., 2003). This compound was a major biomarker observed in the OPLS-DA plot and it has been reported as a major compound of Seville orange peels. **Biomarker 2**: After fragmentation, the peak with a retention time of 6.5 min and at [M-H]^−^ of 595 presented the loss of 308 amu, characteristic of a rutinosyl moiety. The loss of the rutinoside moiety gave a fragmentation ion at 287.1096, indicative of an eriodictyol ion. MS/MS fragmentation of [M − H−136]^−^ at *m/z* 459 is produced by a retro-Diels − Alder reaction in ring C. This biomarker with a UV wavelength of 284 nm was tentatively identified as neoeriocitrin (Mencherini et al., 2013). **Biomarkers 3 and 4**: Compounds 3 and 4 were tentatively identified as flavanone derivatives. Previous research has indicated that the negative product ions at [M H]^−^ = 723 and 753, with a fragment ion of 579 m/z and 609 [M H C6H8O4] indicates the presence of a 3-hydroxy-3-methylglutaryl substituent. Because of the product ions and the UV wavelength of 278 nm, it was assumed that these were a β-hydroxyl β-methylglutary (HMG) conjugate of naringenin and hesperidin, respectively. These compounds were identified as melitidin and bruteiridin, respectively (Brito et al., 2014; Di Donna et al., 2009). **Biomarker 05**: A C-glycosylated compound identified as biomarker 05, presented a pseudomolecular ion at *m/z* 609 and a UV wavelength of 278 nm. The fragmentation observed at *m/*z 489 (M-120), lead to tentative identification as 6,8-di-C-glucoside of luteolin, known as lucenin-2 (Abad-García et al., 2012; Brito et al., 2014).

#### Identification of candidate biomarkers from Eureka lemons

**Biomarker 6** showing a [M-H]^−^ ion at *m/z* 357 was tentatively identified as 3-(2-hydroxy-4-methoxyphenyl)-propanoic acid hexose, as reported by Brito et al. (2014). **Biomarker 7** was tentatively identified as eriocitrin, an isomer of biomarker 01 (Brito et al., 2014). **Biomarkers 8 and 9** were identified as *O*-glucosides- HMG of limocitrol and limocitrin, respectively. These compounds were previously identified by Ledesma-Escobar et al. (2015). In Table 2 a list all the identified candidate biomarkers are shown.

**Table 2:** List of candidate biomarkers for Seville oranges and Eureka lemons detected through multivariate data analysis

Candidate Biomarkers	RT (min)	UV (nm)	MW	[M-H]^−^	Fragmentation pattern	Name	Chemical Formula	References
		**Seville oranges**						
1	7.42	273	580	579.18	12.97, 271.10, 459.18, 581.25, 1159.45	Naringin	C_27_H_32_O_14_	Mencherini et al., (2013)
2	6.54	284	596	595.18	287.10, 459.18, 596.24, 1191.44	Neoeriocitrin	C_27_H_32_O_15_	Guccione et al., (2016)
3	8.46	278	724	723.22	449.21, 579.24, 724.30,	Melitidin	C_33_H_40_O_18_	Brito et al., (2014); Di Donna et al., (2009)
4	8.92	200	754	753.23	609.25, 651	Bruteiridin	C_34_H_42_O_19_	
5	8.07	278	610	609.19	152.64, 489.17, 610.26, 611.26	Lucenin-2	C_27_H_30_O_16_	Brito et al., (2014)
		**Eureka lemons**						
6	5.70	215	358	357.13	195.11, 327.12, 358.17, 358.18, 455.16, 509.25,	3-(2-hydroxy-4-methoxyphenyl)-propanoic acid hexose	C_15_H_22_O_10_	Brito et al., (2014)
7	6.28	225	596	595.17	151.04, 288.11, 287.10, 431.16, 596.24, 597.24, 1191.4,	Eriocitrin (Eriodictyol-7-*O*-rutinose	C_27_H_32_O_15_	Brito et al., (2014)
8	7.89	202	682	681.17	537.19, 651.24, 682.25, 683.25,	Limocitrol-HMG-Glu	C_18_H_16_O_9_	Ledesma-Escobar et al. (2015)
9	7.83	257	652	651.16	301.12, 345.11, 507.18, 652.23, 653.24	Limocitrin-HMG-Glu	C_29_H_32_O_17_	Ledesma-Escobar et al. (2015)

**Fig. 4:**
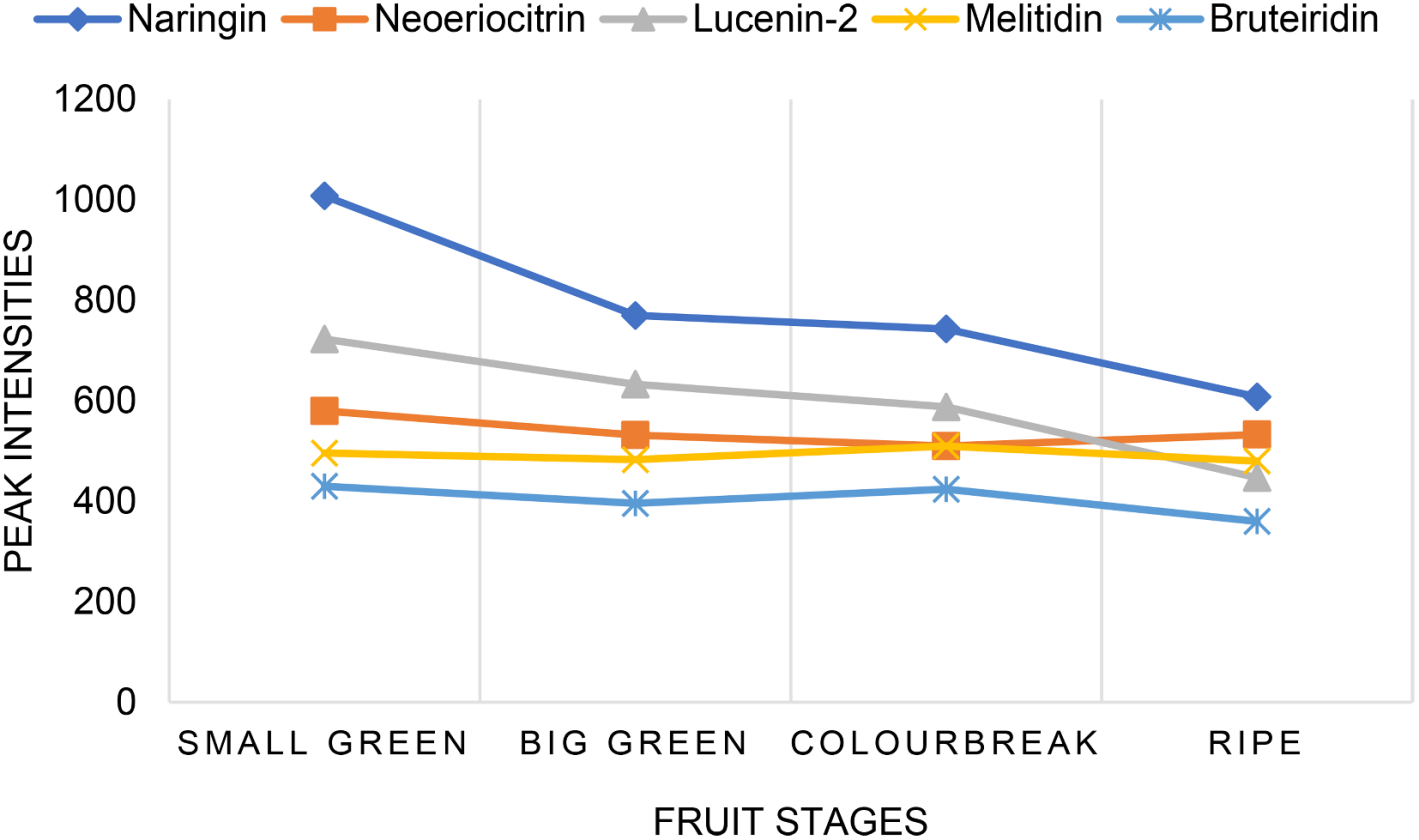
Fruit developmental stage variation in peak intensities of biomarkers identified from Seville peel extracts

The seasonal peak areas of the candidate biomarkers from the tolerant cultivar, Seville orange extracts, represented at different fruit stages are shown in Fig. 4. These candidate biomarkers were found to be high at the beginning of the fruit set when infection by *P. citricarpa* primarily takes place and the accumulation decreases as the fruit develops. A prominent compound in Seville orange peel extracts was found to be naringin, a polymethoxylated flavonoid typically found in citrus fruits, including grapefruit and Seville oranges. Naringin, reported to be responsible for the bitter taste of grapefruit and Seville oranges, is a flavonoid compound with the potential to treat or prevent several ailments, including obesity, heart disease, diabetes, and metabolic syndrome. The medical efficacy of naringin is mostly represented in its antioxidant and anti-inflammatory properties, as well as its pathophysiological protective impact (El-desoukey et al., 2018). Furthermore, naringin has the potential to replace traditional fungicides because it has no known negative effects and several positive aspects to human health compared to that of chemically manufactured fungicides.

### In vitro evaluation of the antifungal activity of naringin, a major candidate biomarker of Seville orange

The antifungal study was tested on naringin only, due to its availability and because it was identified as the major candidate biomarker of Seville orange, the tolerant cultivar. The antifungal efficacy of Demildex, MHTCO_3,_ and cuprous oxide were also evaluated against CBS. The antifungal activities obtained are reported in Table 3.

**Table 3:** Antifungal properties of naringin and selected fungicides against *P. citricarpa*

Compounds/fungicides	Concentrations (mg/L)	% Inhibition
Demildex	2000 mg/L	100%
Cuprous Oxide	18 000 mg/L	100%
MHTCO_3_	10 000 mg/L	100%
Naringin	10 000 mg/L	100%
Naringin	5000 mg/L	60%
Cuprous Oxide + Naringin	5000 mg/L + 4500 mg/L	100%

The negative control using sterile distilled water displayed no inhibitory effect on *P. citricarpa*, while 100% inhibition was observed in the positive control test using Demildex, MHTCO_3_ and cuprous oxide fungicides, at the manufacturers’ recommended concentrations of 2000 mg/L, 11 000 mg/L and 18 000 mg/L, respectively. The in vitro evaluation also indicated that the candidate biomarker, naringin at 10 000 mg/L, was able to completely inhibit the growth of the *P. citricarpa* pathogen. During normal seasons, 18 000 mg/L of cuprous oxide is sprayed twice a year, once in November and once in January. The use of chemical fungicides is common in agricultural practice for field and post-harvest protection. However, restrictions on the application of fungicides have been implemented due to environmental and health concerns. One of the concerns with chemical fungicide is the presence of fungicide residues on the fruit. Similarly, Schutte et al. (2012) observed copper residues during the in vitro evaluation of cuprous oxide application against *P. citricarpa*. Complete inhibition of the pathogen was observed with a combination of naringin and cuprous oxide at 5000 mg/L and 4500 mg/L, respectively. The use of lower cuprous oxide in combination with naringin will be beneficial to the industry by reducing copper residues on the fruit as well its impact on the environment.

## Conclusion

Metabolic profiling of the peel extracts of Seville oranges and Eureka lemons revealed phytochemical differences among these citrus cultivars. The peel extracts of the two cultivars were tested for TSC, and the results indicated that TSC varied across cultivars, fruit stages, and seasons. Total sugar contents increased with fruit maturation, as compared to a decline in phenolic contents as citrus fruit developed. The TSC values of Seville oranges were substantially lower at green stage and might account for the tolerance of this cultivar as CBS infection occurs only when the fruit is very young. The study on the TPC indicated variations in phenol content among the cultivars, seasons, and fruit developmental stages. Seville oranges indicate high phenolic content throughout the seasons and the levels were higher at the beginning of the fruit set, when infections take place. The chemical profile of Seville oranges should be considered when selecting new cultivars.

The multivariate study indicated five candidate biomarkers from Seville oranges and four biomarkers from Eureka lemons. The inhibitory potential of naringin, a major candidate biomarker from Seville orange, the tolerant cultivar was, demonstrated. The positive synergism of the combination of lower concentrations of this candidate biomarker with cuprous oxide provides the industry with the opportunity to reduce copper application in the field.
